# Hypo- and Hypernatremia in Extremely Low Birth Weight Infants in the First 10 Days of Life: A Review

**DOI:** 10.3390/children12020231

**Published:** 2025-02-13

**Authors:** Myrna Pace, Stijn van Sas, Thomas Salaets, Annouschka Laenen, Anke Raaijmakers, Karel Allegaert

**Affiliations:** 1 Faculty of Medicine, KU Leuven, 3000 Leuven, Belgium; myrna.pace@student.kuleuven.be (M.P.); stijn.vansas@student.kuleuven.be (S.v.S.); 2 Pediatric Cardiology, University Hospitals, 3000 Leuven, Belgium; thomas.1.salaets@uzleuven.be; 3 Leuven Biostatistics and Statistical Bioinformatics Center (L-BioStat), KU Leuven, 3000 Leuven, Belgium; annouschka.laenen@kuleuven.be; 4 Department of Paediatric Nephrology, Sydney Children’s Hospital Randwick, Sydney Children’s Hospital Network, Randwick, NSW 2031, Australia; anke.raaijmakers@health.nsw.gov.au; 5 School of Women’s and Children’s Health, Randwick Clinical Campus, University of New South Wales, Randwick, NSW 2033, Australia; 6 Department of Development and Regeneration, KU Leuven, 3000 Leuven, Belgium; 7 Department of Pharmaceutical and Pharmacological Sciences, KU Leuven, 3000 Leuven, Belgium; 8 Department of Hospital Pharmacy, Erasmus MC, 3000 Rotterdam, The Netherlands

**Keywords:** extremely low birth weight, sodium, hyponatremia, hypernatremia

## Abstract

Background/Objectives: Sodium regulation is critical in extremely low birth weight (ELBW, <1000 g) infants. This study aimed to provide a comprehensive overview of sodium dynamics and related variables in ELBW infants in their first 10 days of life through a structured literature review. Methods: Applying PRISMA guidelines, six databases were searched (1 August 2023) on sodium measurements in ELBW cohorts, with quality assessment (RoB2, ROBINS-1, Newcastle Ottawa scale) of retained papers, and subsequent data extraction in line with these PRISMA guidelines to describe findings. Results: Only eight heterogeneous studies could be retained, including observational cohort studies (n = 5), case–control studies (n = 2, Tegaderm application yes/no, gestational age < 24 or 24–28 weeks), and only one randomized trial (sodium restriction versus no sodium restriction). Definitions of hyper- or hyponatremia were also heterogeneous, with incidence ranges for hyper- (8–92.2%) and hyponatremia (0–52.9%). Peak sodium values were observed on days 2–4 in the individual studies. When pooled and compared to the cohort mean sodium values, the highest increases in mean serum sodium values were observed on day 3 (+4, range, −0.6 to +8.6 mEq). Variables of sodium values were related to care factors [incubator settings (open/closed, double-/not double-walled, humidity), fluid regimens (water volume, sodium supplementation), occlusive skin care], as well as related maturational factors (postnatal age, gestational age, small versus appropriate for gestational age, SGA/AGA). Conclusions: Based on a structured literature review, patterns of sodium changes over postnatal age in ELBW cases were documented. Besides incubator settings, fluid regimens, or occlusive skin care, these patterns also depend on maturational factors of the ELBW infant (gestational age, postnatal age, SGA/AGA). These complexities emphasize the need for nuanced interpretation, the relevance of standardizing clinical practices and research definitions, and the need to report on additional datasets.

## 1. Introduction

Despite improvements in survival and outcome, extremely low birth weight (ELBW, <1000 g) infants still face numerous short- and long-term challenges [1,2]. Sodium dysregulation, particularly in the first 10 days of life, poses a significant concern. The incidence, patterns, and reference values of sodium have not yet been reported following a structured review-level analysis of reported findings in this specific ELBW population [3].

Fluid intake and its composition should align with their physiological needs to maintain an equilibrium. Throughout intrauterine development, there is a decrease in extracellular fluid and an increase in intracellular fluid. Driven by maturation, this results in a substantially higher fluid–body ratio and extracellular compartment at birth in preterm compared to term neonates [1]. Postnatally, an abrupt physiological fluid shift occurs, resulting in constriction of the interstitial fluid and the extracellular space [1,2]. To facilitate this, a negative sodium balance is needed in the first 24–48 h of life.

In term infants, this is achieved through a combination of renal sodium loss secondary to renal tubular immaturity and low sodium intake. In preterm infants, these fluid shifts are further modulated due to their kidney and skin immaturity, resulting in a higher likelihood of developing hypernatremia. As nephron development correlates closely with gestational age, ELBW infants have a lower nephron number and glomerular filtration rate, reflecting renal immaturity [2]. Additionally, transepidermal water loss is increased due to the relatively high body surface-area-to-weight ratio and their not yet keratinized, and therefore more permeable skin. Consequently, hypo- or hypernatremia is more likely to occur in ELBW infants. On the one hand, hypernatremic dehydration poses risks, including cerebral insults and its association with severe hyperbilirubinemia [4]. On the other hand, excessive fluid (free water) supplementation and hyponatremia increase the risk of patent ductus arteriosus (PDA), necrotizing enterocolitis (NEC) and bronchopulmonary dysplasia (BPD) [1,2,5,6,7].

The lack of reference sodium values and standardized definitions for hypo- and hypernatremia for this population further complicates fluid supplementation guidance and interpretation of individual sodium observations or trends over postnatal life in a given ELBW newborn [8,9]. It also makes clinical studies on, e.g., associations between hypo- or hypernatremia and short- or long-term outcomes more difficult to pool and understand. This study therefore aimed to evaluate the serum sodium concentration pattern and range in ELBW infants in the initial 10 days after birth, along with associated variables, as well as the shortages related to the currently available information, or standardization of definitions. To do so, we conducted a structured literature review of the existing data on sodium values and related variables.

## 2. Materials and Methods

### 2.1. Structured Literature Review

A structured literature review was conducted following PRISMA guidelines [10]. We searched for relevant articles across PubMed, Embase, Web of Science Core Collection, Scopus, Cochrane Library, and CINAHL (all searches conducted on 1 August 2023). The search strategy is provided as a Supplement (Table S1). This review was registered on PROSPERO (CRD42023446950) on 27 July 2023. Ethics approval for this study was provided by the ethics board of KU Leuven, Belgium (MP024264). We selected articles published in English, French and Dutch. Inclusion criteria required studies on ELBW infants with serial serum sodium measurements from birth. Studies on non-ELBW infants, lacking sodium measurements, or without full texts were excluded, as were editorials and reviews. No timeframe restrictions were imposed. Two authors (M.P., S.v.S.) developed search terms and entries for the six databases and independently selected articles, supported by librarian experts (Krizia Tuand, Chayenne van Meel).

**Figure 1:**
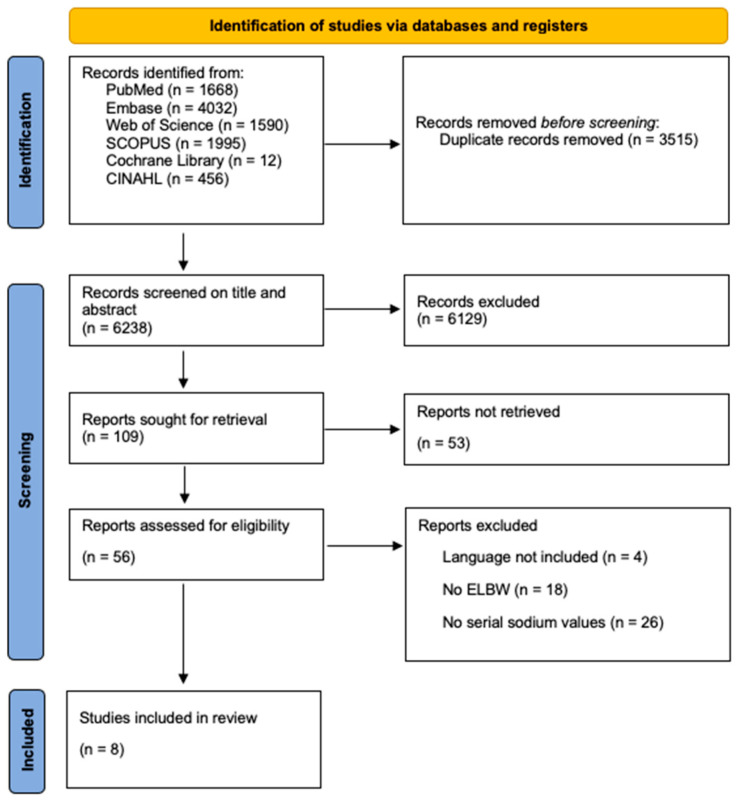
Prisma 2020 flow diagram.

First, titles and abstracts were screened, followed by full-text analysis in the second round, as outlined in the selection process shown in the PRISMA flow diagram in Figure 1, using the Rayyan platform [10,11]. Included studies underwent an independent quality assessment by two reviewers (M.P., S.v.S.) (Tables S2 and S3). We hereby used the RoB2 tool for randomized controlled trials, the ROBINS-1 tool for non-randomized controlled trials and the Newcastle Ottawa scale for cohort studies [12,13,14]. Throughout the process of article selection and quality assessment, the authors (M.P. and S.v.S.) resolved disagreements through discussion or by consulting a third reader (K.A.).

### 2.2. Data Handling

Daily sodium values from each study were obtained either from reported data in text or tables or by using WebPlotDigitizer 4.6 to extract data from figures [15,16]. Key study characteristics were summarized, including the study design, gestational age and birth weight and being small for gestational age (SGA, birth weight below the 10th percentile for gestational age) or appropriate for gestational age (AGA, birth weight between the 10th and 90th percentile for gestational age). Furthermore, information on cutoff values for hypo- and hypernatremia as defined in the specific study, incidence of hypo- and hypernatremia, complications, specific interventions, incubator settings and fluid regimens were extracted.

## 3. Results

### 3.1. Structured Literature Review

We identified 9753 studies in the aforementioned databases. After removing duplicates and applying inclusion and exclusion criteria, eight studies were retained (Figure 1). The results of the quality assessment are provided in the Supplemental Materials (Tables S2 and S3), while the overall risk of bias in the studies appeared to be low.

### 3.2. Study Characteristics

Table 1 summarizes the characteristics of included studies and the associations of various interventions or characteristics on the serum sodium levels in ELBW neonates. Studies varied extensively in design (observational cohort studies = 5; case–control studies = 2, Tegaderm application yes/no, gestational age < 24 or 24–28 weeks; randomized trial, n = 1; sodium restriction versus no sodium restriction), sample size (12–12.428 neonates), gestational age (22.9–28.9 weeks), birth weight (493.4–850 g) and follow-up periods (5–14 days). Common exclusion criteria were congenital anomalies, severe infections, persistent ductus arteriosus, acute kidney injury and death or transfer within the first week of life (trial-specific exclusion criteria are provided in Table 1).

Table 2 summarizes cutoff values, incidences and variables associated with serum sodium levels in ELBW neonates.

We also noticed heterogeneity in the definitions applied. Hypernatremia cutoffs between the different studies ranged from >145 to >150 mEq/L, with incidences between 8% and 92.2%, while hyponatremia cutoffs were typically <130 mEq/L, occasionally set as <125 mEq/L with incidences between 0 and 52.9%. Peak sodium values were observed on days 2–4.

Factors related to the sodium patterns in ELBW patients were in part care-related [incubator settings (open/closed, double-/not double-walled, humidity), fluid regimens (water volume, sodium supplementation), occlusive skin care] and in part related to maturational factors (postnatal age, gestational age, small versus appropriate for gestational age, SGA/AGA).

**Figure 2:**
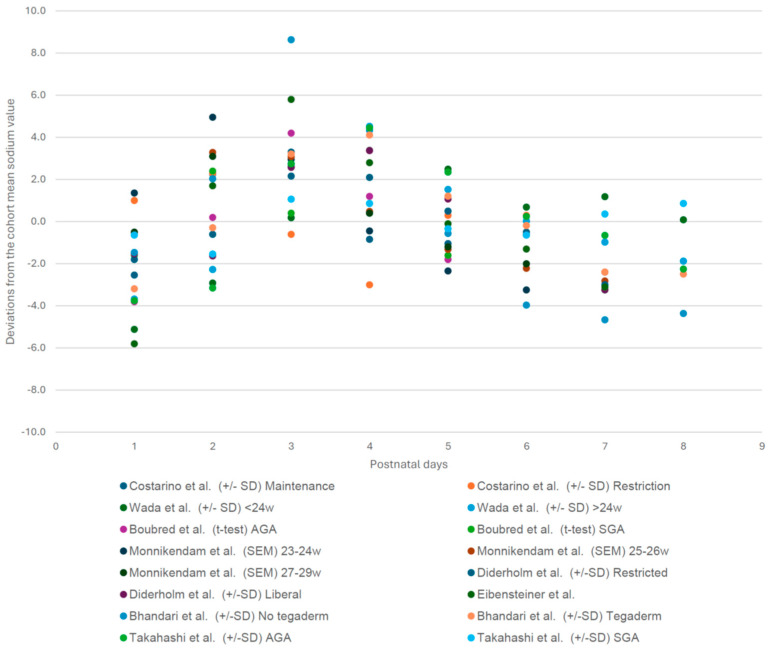
Sodium pattern extracted from the included studies, shown as deviations from the cohort mean daily sodium value for each data group on the *Y*-axis. *X*-axis: days after birth. This figure shows the consistent pattern of changes in sodium values, regardless of the height of the mean sodium values in each cohort, as these somewhat varied secondary to the different treatment regimens and interventions [1,3,7,17,18,19,20,21].

The studies showed a similar trend in the serum sodium concentration of ELBW infants, with a rise after birth that peaked on days 2–4, followed by a decrease to steady-state values as illustrated in Figure 2. Unfortunately, because of the way the data on sodium values were reported in subgroups in the majority of the individual papers, we were unable to extract and provide data on mean sodium values over postnatal age.

### 3.3. Cohort Specific Sodium Deviation Pattern of Each Postnatal Day (1–8) Compared to the Cohort Mean Sodium Value [1,3,7,17,18,19,20,21]

Finally, the studies were also quite heterogeneous in their study design and research questions. Despite this, most studies found significant differences between groups or conditions that were compared. Costarino et al. found that sodium restriction led to lower serum sodium concentration than daily maintenance replacement from day 2 to day 5 (*p* < 0.001) [7]. Wada et al. found that gestational age did not influence serum sodium concentrations until day 3, but after that, higher serum sodium concentrations were associated with lower gestational age [1]. Bhandari et al. found that Tegaderm^®^ application reduced serum sodium concentrations compared to no Tegaderm (*p* = 0.031) [20]. Takahashi et al. found that AGA infants had significantly higher serum sodium concentrations than SGA infants on days 3 and 4, while Boubred et al. did not find a significant difference between both groups [17,21]. Diderholm et al. did not find a significant difference in serum sodium concentrations between a restrictive or liberal fluid regimen [18]. The study by Monnikendam et al. showed that the mean sodium values of 23–24-week-old infants were significantly higher than those of 25–26-week-old infants on days 1–3 and 7. The 27–29-week cohort had significantly lower mean sodium values than the other two cohorts on every day of the 7-day period [3].

## 4. Discussion

Sodium regulation is of the utmost importance, especially for ELBW infants. This structured review provides an overview of the observed sodium values in ELBW infants during the first ten days of life. We hereby were struck by a limited number of studies and the heterogeneous characteristics in both study design and the definitions applied.

This structured review reveals an initial increase in sodium levels facilitated by a physiological fluid shift in the first 24–48 h of life, reaching a peak on day 3, followed by a gradual decrease [5,6]. However, besides the patterns, it is important to realize that different factors, either related to care practices or maturational factors, affect sodium values.

Different care practices were used in the different observational studies, while we could only retrieve one prospective RCT (sodium restriction versus daily maintenance replacement) [7]. The variables affecting sodium values were related to incubator settings (open/closed, double-/not double-walled, humidity), fluid regimens (water volume, sodium supplementation) and occlusive skin care. From a research perspective, we noticed that different cutoffs were used to define hyper- and hyponatremia (Table 1) resulting in varying incidences between studies. These findings emphasize the need to establish reference values, driven by relevant patient outcome variables [8].

ELBW infants experience prolonged water loss, necessitating fluid and sodium supplementation after initial weight loss [5,22]. Strategies like near 100% humidification and Tegaderm patches proved to be effective in minimizing transepidermal water loss and reducing hypernatremia incidence, secondary to reduced water losses [1,4,17,23]. The challenge lies in balancing the need for fluids to prevent hypernatremia, dehydration and hyperbilirubinemia while avoiding potential complications of excessive fluid administration such as PDA, BPD and NEC. Ideally, fluid supplementation allows contraction of the extracellular space and weight loss but helps avoid early hypernatremia and provides enough sodium necessary for growth after the first few days [22]. Both Eibensteiner and Costarino showed that restricting the sodium intake resulted in better outcomes [7,19]. Costarino proved that a decrease in sodium intake caused significantly less BPD [7]. Eibensteiner proved that it reduces BPD incidence, higher-grade intraventricular hemorrhage (IVH), NEC and mortality (*p* < 0.01) [19].

Besides the above listed ‘care process’ related factors, maturational factors (gestational age, postnatal age, SGA/AGA) co-determine the sodium observation patterns. Gestational age’s association with sodium values varied across the first 10 days in the case of infants aged <24 weeks, compared to the 24–28-week-old controls [1]. A separate study by Monnikendam et al. revealed that infants with a higher gestational age had lower serum sodium values [3]. Stritzke et al. analyzed cord blood sodium levels in over 500 neonates, uncovering a trend of lower averages in preterm infants, with sodium values increasing with maturity [24]. We assume that cord blood largely correlates with serum sodium levels on the first day of life. The decrease in sodium values with decreasing gestational age observed in Strizke et al.’s research aligns with the findings from our own cohort on day 1 of life.

A higher birth weight was associated with lower average sodium values. Monnikendam et al.’s data indicated an association between moderate and severe hypernatremia and lower birth weight and gestational age [3]. Finally, Boubred et al. SGA infants showed a trend towards lower serum sodium values compared to AGA infants, although there was no statistical significance [17].

Our study has limitations, reflecting the shortages in the current research field. The large heterogeneity among the analyzed studies requires a cautious interpretation of the pooled data, while future research should be aimed at uncovering normative values, standardizing definitions and optimizing fluid and sodium supplementation protocols. Because of the way the data on sodium values were reported in the individual papers, we could not provide data on mean sodium values over postnatal age. This also makes clinical studies on, e.g., associations between hypo- or hypernatremia and short- or long-term outcomes more difficult to pool and understand.

In conclusion, we provided sodium trends and reference values over postnatal age in ELBW cases, while new variables of this sodium pattern were suggested. While associations between serum sodium levels and gestational age as well as birth weight and birth length are in line with what has been previously described in the literature, we add the association between cesarean sections, inotropic agents, ibuprofen use and serum sodium values in ELBW infants to this list of associated variables. This should be further prospectively validated and confirmed in larger cohorts. Such reference values are useful to provide clinicians guidance to interpret single sodium observations or postnatal trends in individual ELBW newborns.

## Figures and Tables

**Table 1:** General characteristics of the eight included studies that examined the associations of various interventions or variables on serum sodium levels of ELBW neonates.

Author	Study Design	Groups	Number of Neonates Included	Weeks of Gestation at Birth	Birth Weight	Follow Up Period	Exclusion Criteria
Costarino et al. [7]	Randomized controlled trial	Sodium restriction	9	27 ± 1 weeks	870 ± 120 g	5 days	Renal malformation, renal arterial or venous thrombosis, Apgar score of <5 at 5 min.
		No sodium restriction	8	27 ± 1 weeks	820 ± 130 g		
Wada et al. [1]	Retrospective case–control	Cases	17	22.9 ± 0.6 weeks	493.4 ± 66.2 g	7 days	Congenital anomalies, severe infections or symptomatic persistent ductus arteriosus.
		Controls	72	25.6 ± 0.7 weeks	777.8 ± 99.1 g		
Boubred et al. [17]	Retrospective cohort study	AGA	36	25.1 ± 0.9 weeks	837 ± 146 g	5 days	On the national growth curves for children. Congenital malformations or severe illness requiring medication that could influence the electrolyte balance, such as insulin, NSAIDs, diuretics. Infants who died during the study period. Lacking laboratory variables on days 1–4.
		SGA	12	25.9 ± 1.1 weeks	665 ± 109 g		
Monnikendam et al. [3]	Retrospective cohort study		12,428	N/A	N/A	7 days	Significant congenital anomalies, death/transfer within 7 days of birth or missing daily sodium in the first 7 days of life leaving.
Diderholm et al. [18]	Retrospective cohort study	Restricted fluid intake	63	25.2 ± 1.2 weeks	744 ± 192 g	5 days	Died or transferred to other units within the first week of life.
		Liberal fluid intake	112	25.1 ± 1.1 weeks	718 ± 156 g		
Eibensteiner et al. [19]	Retrospective cohort study		94 included, 90 analyzed	Mean: 24 + 6/7 weeks	718 g	14 days	Death within 24 h of birth, acute kidney injury (serum creatinine > 1.5 mg/dL), syndrome of inappropriate ADH secretion, adrenogenital syndrome, diabetes insipidus.
Bhandari et al. [20]	Retrospective case–control	Pre-Tegaderm	39	26.1 ± 1.9 weeks	756 ± 158 g	7 days	N/A
		Tegaderm	30	26.3 ± 1.8	802 ± 160 g		
Takahashi et al. [21]	Retrospective cohort study	AGA	72	25.9 ± 1.7 weeks	787.35 ± 148.5 g	7 days	Died, had PDA, intestinal obstruction and severe infection.
		SGA	28	28.9 ± 2.6 weeks	762.2 ± 156.3 g		

**Table 2:** Overview of the incidence of hyponatremia, hypernatremia, highest sodium values, incubator settings and fluid regimen for the eight included studies.

Author		Cutoff for Hyponatremia	Cutoff for Hypernatremia	Incidence of Hyponatremia	Incidence of Hypernatremia	Peak Sodium	Incubator Settings	Fluid Regimen
Costarino et al. [7]	Sodium restriction	<130 mEq/L	>150 mEq/L	22.22%	0%	Day 2	Radiant warmers + transparent plastic blankets.	3–4 mEq/kg/day of sodium, volume of fluid determined by physician.
	No sodium restriction			0%	25%	Day 2		Maintenance sodium supplementation, volume of fluid determined by physician.
Wada et al. [1]	Cases	<130 mEq/L	>150 mEq/L	17.70%	29%	Day 4	Closed incubator, near 100% humidity day 0–3, then highly humidified environment >80%.	Solution of 7.5% glucose mixed with calcium gluconate in the ratio of 19:1 (or 18:2) at 50 mL/kg per day.
	Controls			26.40%	9.70%	Day 4		
Boubred et al. [17]	AGA	N/A	N/A	N/A	N/A	Day 3	N/A	Day 1: total parenteral nutrition provided glucose (6 g/kg/day), protein (2 g/kg/day) and lipid (1 g/kg/day. Progressively increased until they reached 4 g/kg/day protein and 3 g/kg/day lipids by the end of the first week.
	SGA			N/A	N/A	Day 2		
Monnikendam et al. [3]		125–134 mEq/L, <125 mEq/L for severe	145–154 mEq/L, >154 mEq/L for severe	<135: 35.1%	145–154: 65.4%, >154 21.7%	Day 2	N/A	N/A
Diderholm et al. [18]	Restricted fluid intake	<130 mmol/L	>145 mmol/L moderate, >150 mmol/L severe	21%	>145: 29%, >150: 10%	Day 3	First week: humidity of 85%, after: humidity of 50%	Total fluids 95 mL/kg/day (GA 22–24 weeks) and 85 mL/kg/d (GA 25–26 weeks). Increase in fluid with 10 mL/kg/d, to be adjusted at the discretion of the attending neonatologist.
	Liberal fluid intake			21%	>145: 36%, >150: 11%	Day 4		
Eibensteiner et al. [19]		N/A	>145 mEq/L: mild, >150 mEq/L: severe	N/A	>145: 92.2%, >150: 64.4%	Day 3	Double-wall incubators with humidity 85–90%, humidity was reduced by 5%/week.	N/A
Bhandari et al. [20]	No Tegaderm	N/A	> 150 mEq/L	N/A	51%	Day 3	Initially in radiant warmers, incubators when clinically stable.	N/A
	Tegaderm			N/A	17%	Day 4		
Takahashi et al. [21]	AGA	<130 mEq/L	>150 mEq/L	27.59%	8.62%	Day 4	Almost full ambient humidity in closed incubator with nebulizer until 4th day of life, 90% humidity without nebulizer after that.	50–60 mL/kg on first day of life.
	SGA			52.94%	5.88%	Day 3		

## Data Availability

The data collected are already in the public domain.

## Supplementary Materials

The following supporting information can be downloaded at: https://www.mdpi.com/article/10.3390/children12020231/s1, Table S1. Search entries in Pubmed, Embase, Web of Science Core Collection, Scopus, Central (via Cochrane Library) and CINAHL; Table S2. Quality assessment of randomized and non-randomized controlled trials using the ROBINS-I and RoB 2 tool [12,13]; Table S3. Quality assessment of cohort studies organized by date using the Newcastle Ottawa Scale (NOS) tool containing four domains of selection, one domain of comparability and three domains of exposure [14].
