# Supplementary material for: Hypo- and Hypernatremia in Extremely Low Birth Weight Infants in the First 10 Days of Life: A Review
Source: Children (Basel). 2025 Feb 13;12(2):231. doi: 10.3390/children12020231 (PMC11854672; doi:10.3390/children12020231)
Supplement: Supplementary file 1 [file children-12-00231-s001.zip › children-3310309-supplementary.pdf]

Supplemental material

Table S1. Search entries in Pubmed, Embase, Web of Science Core Collection, Scopus, Central (via Cochrane Library) and CINAHL.

Table S2. Quality assessment of randomized and non-randomized controlled trials using the ROBINS-I [12] and RoB 2 [13] tool.

Table S3. Quality assessment of cohort studies organized by date using the Newcastle Ottawa Scale (NOS) tool containing four domains of selection, one domain of comparability and three domains of exposure [14].

Table S1: Search entries in Pubmed, Embase, Web of Science Core Collection, Scopus, Central (via Cochrane Library) and CINAHL.

PubMed	("Infant, Low Birth Weight"[Mesh] OR "Extremely Low Birth Weight*"[tiab] OR "Extremely Low Birthweight*"[tiab] OR “LBW”[tiab] OR “VLBW”[tiab] OR “ELBW”[tiab] OR "Infant, Premature"[Mesh] OR “Premature Infant*”[tiab] OR “Premature bab*”[tiab] OR “Premature Newborn*”[tiab] OR “Premature Neonate*”[tiab] OR “Preterm Baby*”[tiab] OR “Preterm Infant*”[tiab] OR “Preterm Newborn*”[tiab] OR “Preterm Neonate*”[tiab] OR “Neonatal Prematurity”[tiab] OR "Intensive Care Units, Neonatal"[Mesh] OR “neonatal intensive care unit*”[tiab] OR "Neonatal ICU*"[tiab] OR "Newborn Intensive Care Unit*"[tiab] OR "Newborn ICU*"[tiab]) AND ("Sodium"[Mesh:NoExp] OR "Sodium"[tiab] OR "Hypernatremia"[Mesh] OR "Hypernatr*"[tiab] OR "Hyponatremia"[Mesh] OR "Hyponatr*"[tiab] OR “Dysnatr*”[tiab] OR "Water-Electrolyte Balance"[Mesh] OR "Water-Electrolyte Imbalance"[Mesh:NoExp] OR "WaterElectrolyte Imbalance"[tiab] OR "Water Electrolyte Balance"[tiab] OR "Electrolyte Balance"[tiab] OR "Water Electrolyte Imbalance*"[tiab])
Embase	'low birth weight'/exp OR ‘low birth weight*’:ti,ab,kw OR ‘low birthweight*’:ti,ab,kw OR 'LBW':ti,ab,kw OR 'VLBW':ti,ab,kw OR 'ELBW':ti,ab,kw OR 'prematurity'/exp OR ‘Premature neonate’:ti,ab,kw OR ‘Premature Infant*’:ti,ab,kw OR ‘Premature bab*’:ti,ab,kw OR ‘Premature Newborn*’:ti,ab,kw OR ‘Preterm Bab*’:ti,ab,kw OR ‘Preterm Infant*’:ti,ab,kw OR ‘Preterm Newborn*’:ti,ab,kw OR ‘Preterm Neonate*’:ti,ab,kw OR ‘Neonatal Prematurity’:ti,ab,kw OR 'neonatal intensive care unit'/exp OR 'NICU*':ti,ab,kw OR 'neonatal intensive care unit*':ti,ab,kw OR 'Neonatal ICU*':ti,ab,kw OR 'Newborn Intensive Care Unit*':ti,ab,kw OR 'Newborn ICU*':ti,ab,kw) AND ('sodium'/exp OR 'sodium':ti,ab,kw OR 'natrium':ti,ab,kw OR 'hypernatremia'/exp OR 'hypernatr*':ti,ab,kw OR 'hyponatremia'/exp OR 'hyponatr*':ti,ab,kw OR 'dysnatremia'/exp OR 'dysnatr*':ti,ab,kw OR 'electrolyte balance'/exp OR 'electrolyte disturbance'/exp OR 'electrolyt*’:ti,ab,kw)
Web of Science Core Collection	(TS=(“low birth weight” OR “low birth weight*” OR “low birthweight*” OR “LBW” OR “VLBW” OR “ELBW” OR “prematurity” OR “Premature neonate” OR “Premature Infant*” OR “Premature bab*” OR “Premature Newborn*” OR “Preterm Bab*” OR “Preterm Infant*” OR “Preterm Newborn*” OR “Preterm Neonate*” OR “Neonatal Prematurity” OR “neonatal intensive care unit”/exp OR “NICU*” OR “neonatal intensive care unit*” OR “Neonatal ICU*” OR “Newborn Intensive Care Unit*” OR “Newborn ICU*”)) AND (TS=(“sodium” OR “sodium” OR “natrium” OR “hypernatremia” OR “hypernatr*” OR “hyponatremia” OR “hyponatr*” OR “dysnatremia” OR “dysnatr*” OR “electrolyte balance” OR “electrolyte disturbance” OR “electrolyt*”))
Scopus	(TITLE-ABS(“low birth weight” OR “low birth weight*” OR “low birthweight*” OR “LBW” OR “VLBW” OR “ELBW” OR “prematurity” OR “Premature neonate” OR “Premature Infant*” OR “Premature bab*” OR “Premature Newborn*” OR “Preterm Bab*” OR “Preterm Infant*” OR “Preterm Newborn*” OR “Preterm Neonate*” OR “Neonatal Prematurity” OR “neonatal intensive care unit”/exp OR “NICU*” OR “neonatal intensive care unit*” OR “Neonatal ICU*” OR “Newborn Intensive Care Unit*” OR “Newborn ICU*”)) AND (TITLE-ABS(“sodium” OR “sodium” OR “natrium” OR “hypernatremia” OR “hypernatr*” OR “hyponatremia” OR “hyponatr*” OR “dysnatremia” OR “dysnatr*” OR “electrolyte balance” OR “electrolyte disturbance” OR “electrolyt*”))

Cochrane Library	#1: [mh "Infant, Low Birth Weight"] OR [mh "Infant, Premature"] OR [mh "Intensive Care Units, Neonatal"]<br>#2: ((“Low Birth” NEXT Weight*) OR (“Low” NEXT Birthweight*) OR "LBW" OR "VLBW" OR "ELBW" OR ((preterm* OR pre-term* OR prematur*) NEXT (Infant* OR Bab* OR Newborn* OR Neonate*)) OR (“newly born” NEXT child*) OR (“newly born” NEXT infant*) OR NICU*OR neonatal intensive care unit*OR Neonatal ICU* OR Newborn Intensive Care Unit* OR Newborn ICU*):ti,ab,kw<br>#3: [mh ^"Sodium"] OR [mh "Hypernatremia"] OR [mh "Hyponatremia"] OR [mh "WaterElectrolyte Balance"] OR [mh ^"Water-Electrolyte Imbalance"]<br>#4: ("Sodium" OR Hypernatr* OR Hyponatr* OR Dysnatr* OR "Water-Electrolyte Imbalance" OR "Water Electrolyte Balance" OR "Electrolyte Balance” OR ("Water Electrolyte” NEXT Imbalance*)):ti,ab,kw<br>#5: #1 OR #2<br>#6: #3 OR #4<br>#7: #5 AND #6
CINAHL	((MH “Infant, Low Birth Weight+”) OR (MH "Infant, Premature") OR (MH "Intensive Care Units, Neonatal") OR (MH "Intensive Care, Neonatal") OR (MH "Neonatal Intensive Care Nursing") OR (MH "Neonatal Intensive Care Nurses") OR TI(“low birth weight” OR “low birth weight*” OR “low birthweight*” OR “LBW” OR “VLBW” OR “ELBW” OR “prematurity” OR “Premature neonate” OR “Premature Infant*” OR “Premature bab*” OR “Premature Newborn*” OR “Preterm Bab*” OR “Preterm Infant*” OR “Preterm Newborn*” OR “Preterm Neonate*” OR “Neonatal Prematurity” OR “neonatal intensive care unit”/exp OR “NICU*” OR “neonatal intensive care unit*” OR “Neonatal ICU*” OR “Newborn Intensive Care Unit*” OR “Newborn ICU*”) OR AB(“low birth weight” OR “low birth weight*” OR “low birthweight*” OR “LBW” OR “VLBW” OR “ELBW” OR “prematurity” OR “Premature neonate” OR “Premature Infant*” OR “Premature bab*” OR “Premature Newborn*” OR “Preterm Bab*” OR “Preterm Infant*” OR “Preterm Newborn*” OR “Preterm Neonate*” OR “Neonatal Prematurity” OR “neonatal intensive care unit”/exp OR “NICU*” OR “neonatal intensive care unit*” OR “Neonatal ICU*” OR “Newborn Intensive Care Unit*” OR “Newborn ICU*”)) AND ((MH "Hypernatremia") OR (MH "Electrolyte Management: Hypernatremia (Iowa NIC)") OR (MH "Hyponatremia") OR (MH "Electrolyte Management: Hyponatremia (Iowa NIC)") OR TI(“sodium” OR “sodium” OR “natrium” OR “hypernatremia” OR “hypernatr*” OR “hyponatremia” OR “hyponatr*” OR “dysnatremia” OR “dysnatr*” OR “electrolyte balance” OR “electrolyte disturbance” OR “electrolyt*”) OR AB(“sodium” OR “sodium” OR “natrium” OR “hypernatremia” OR “hypernatr*” OR “hyponatremia” OR “hyponatr*” OR “dysnatremia” OR “dysnatr*” OR “electrolyte balance” OR “electrolyte disturbance” OR “electrolyt*”))

Table S2. Quality assessment of randomized and non-randomized controlled trials using the ROBINS-I [12] and RoB 2 [13] tool.

	D1	D2	D3	D4	D5	D6	D7	D8
Costarino et al, 1992 [5]			+	+	+	+	?	+
Bhandari et al, 2005 [6]	+	+		+	?	+	+	+

Domains:

D1 Bias due to confounding

D2 Bias in selection of participants into the study

D3 Randomization process

D4 Bias due to derivations from intended interventions

D5 Bias due to missing data

D6 Bias in measurements of the outcome

D7 Bias in selection of the reported results

D8 Overall bias

Risk of bias:

+ Low risk

? Some concerns

- High risk

Table S3. Quality assessment of cohort studies organized by date using the Newcastle Ottawa Scale (NOS) tool containing four domains of selection, one domain of comparability and three domains of exposure [14].

**Study**	**Author**	Wada et al.[1]	Boubred et al.[17]	Monnikendam et al. [3]	Eibensteiner et al. [19]	Takahashi et al.[21]	Diderholm et al. [18]
	**Year**	2008	2015	2019	2020	1993	2022
**Selection**	Representativeness of exposed cohort	*	*	*	*	*	*
	Selection of the non-exposed cohort		*	*	*	*	*
	Ascertainment of the exposure	*	*	*	*	*	*
	Demonstration that outcome of interest was not present at start of study		*				*
**Comparability**	Comparability of cohorts on the basis of the design or analysis	**	**	**	**	**	*
**Outcome**	Assessment of outcome	*	*	*	*	*	*
	Was follow up long enough for outcomes to occur?	*	*	*	*	*	*
	Adequacy of follow up for cohorts	*	*	*	*	*	*
**Stars**		7	9	8	8	8	8
